# Metformin and Hypertensive Disorders of Pregnancy: A Phenotype-Based Focused Narrative Review

**DOI:** 10.3390/jcm15166423

**Published:** 2026-08-19

**Authors:** Katarina Ivanovic, Stefan Dugalic, Miroslava Gojnic Dugalic, Milos Milincic

**Affiliations:** 1 Clinic for Gynecology and Obstetrics, University Clinical Centre of Serbia, 11000 Belgrade, Serbia; stef.dugalic@gmail.com (S.D.); miroslavagojnicdugalic@yahoo.com (M.G.D.); milosmilincic@gmail.com (M.M.); 2 Faculty of Medicine, University of Belgrade, 11000 Belgrade, Serbia

**Keywords:** metformin, pregnancy, preeclampsia, gestational hypertension, hypertensive disorders of pregnancy, gestational diabetes mellitus, type 2 diabetes mellitus, obesity, polycystic ovary syndrome, placental dysfunction

## Abstract

**Background:** Hypertensive disorders of pregnancy (HDP), particularly gestational hypertension and preeclampsia, are major causes of maternal and perinatal morbidity. Metabolic disorders such as gestational diabetes mellitus (GDM), type 2 diabetes mellitus (T2DM), obesity, and polycystic ovary syndrome (PCOS) share mechanisms with HDP, including insulin resistance, inflammation, oxidative stress, endothelial dysfunction, and placental maladaptation. Metformin may influence these pathways, but its clinical effect on hypertensive outcomes remains uncertain. **Methods:** A focused narrative review was conducted using structured, targeted searches of PubMed/MEDLINE, Scopus, Web of Science, and Google Scholar for literature published primarily from January 2015 through March 2026, supplemented by landmark studies, clinical guidelines, randomized trials, large observational cohorts, systematic reviews, meta-analyses, and mechanistic and placental studies. Evidence was selected for its relevance to the predefined phenotype-based framework and synthesized qualitatively across mechanistic, clinical, and translational domains. **Results:** Metformin improves insulin sensitivity and may reduce gestational weight gain, metabolic stress, inflammatory signaling, and oxidative stress. Some randomized trials and meta-analyses in GDM, obesity, and PCOS suggest lower rates of pregnancy-induced hypertension or preeclampsia, whereas recent randomized and population-based studies report neutral effects. Findings are limited by heterogeneous populations, late treatment initiation, variable comparators, supplemental insulin use, inconsistent HDP definitions, and insufficient statistical power. **Conclusions:** Metformin may act as a metabolic and vascular modifier in selected pregnancies, but current evidence does not support its routine use specifically for HDP prevention. Its public health value lies primarily in accessible metabolic treatment and integration into comprehensive antenatal and postpartum risk reduction pathways, rather than replacement of aspirin, blood pressure surveillance, or maternal–fetal monitoring.

## 1. Introduction

Hypertensive disorders of pregnancy (HDP), including chronic hypertension, gestational hypertension, preeclampsia, and chronic hypertension with superimposed preeclampsia, are among the most important causes of maternal and perinatal morbidity and mortality worldwide [1,2]. Preeclampsia may progress rapidly from new-onset hypertension to multisystem maternal organ dysfunction and is associated with placental dysfunction, fetal growth restriction, medically indicated preterm birth, and increased perinatal risk [2,3]. The clinical significance of HDP extends beyond the index pregnancy, because affected women have an increased long-term risk of chronic hypertension, ischemic heart disease, heart failure, stroke, and metabolic disease [1,2,4].

Maternal metabolic health is closely related to hypertensive risk during pregnancy. Overweight and obesity are associated with progressively higher risks of gestational hypertension and preeclampsia, while gestational diabetes mellitus (GDM), pregestational type 2 diabetes mellitus (T2DM), and polycystic ovary syndrome (PCOS) frequently coexist with obesity, insulin resistance, dyslipidemia, and chronic low-grade inflammation [5,6]. Women with PCOS also have higher risks of GDM, gestational hypertension, and preeclampsia, even in analyses accounting for age and body mass index [6]. These overlapping cardiometabolic phenotypes have generated interest in whether therapies targeting insulin resistance might provide vascular benefits beyond glycemic control.

Metformin is increasingly used during pregnancy for GDM, T2DM, and selected women with PCOS. Interest in metformin as a potential modifier of hypertensive risk has been driven by randomized trials and meta-analyses reporting lower gestational weight gain and possible reductions in pregnancy-induced hypertension or preeclampsia compared with insulin, placebo, or other treatments [7,8]. However, the apparent effect varies according to maternal indication and comparator. In the meta-analysis by Kalafat et al., metformin was associated with a lower risk of pregnancy-induced hypertension than insulin in women with GDM, whereas the reduction in preeclampsia was not statistically significant and evidence in women with obesity was heterogeneous [7]. A broader meta-analysis reported a modest reduction in preeclampsia across different maternal indications, but also identified substantial clinical heterogeneity and increased gastrointestinal adverse effects [8].

More recent evidence has challenged the interpretation of metformin as an HDP-preventive therapy. A population-based analysis combining national data from Scotland and Sweden found no association between metformin exposure and either composite HDP or preeclampsia among women with diabetes after adjustment for measured confounders [9]. Similarly, the contemporary SUGAR-DIP randomized trial did not demonstrate noninferiority of a sequential oral glucose-lowering strategy initiated with metformin compared with insulin for its primary perinatal outcome, while pregnancy-induced hypertension, preeclampsia, and most other secondary outcomes did not differ materially between treatment groups [10]. These findings indicate that improvements in gestational weight gain, glycemic control, or treatment burden cannot automatically be interpreted as prevention of placenta-mediated disease.

Biological plausibility nevertheless remains relevant. Insulin resistance, hyperinsulinemia, dyslipidemia, oxidative stress, inflammatory activation, endothelial dysfunction, mitochondrial disturbance, and placental maladaptation contribute in varying degrees to GDM, T2DM, obesity, PCOS, and HDP [3,5,6]. Metformin influences several of these pathways, including hepatic glucose production, insulin sensitivity, cellular energy sensing, mitochondrial activity, inflammatory signaling, and endothelial function. However, preeclampsia is biologically heterogeneous: early-onset disease is more strongly associated with abnormal placentation and angiogenic imbalance, whereas late-onset disease may more prominently reflect the interaction between placental demand and maternal cardiovascular or metabolic vulnerability [2,3]. A metabolically active intervention may therefore have different effects across HDP phenotypes and may be more likely to modify maternal metabolic and cardiovascular adaptation than an established early placental lesion.

Interpretation of the available literature is further complicated by differences in maternal phenotype, timing and duration of exposure, metformin dose, comparator therapy, supplemental insulin use, aspirin prophylaxis, baseline renal and vascular disease, treatment response, and definitions of hypertensive outcomes [7,8,9,10]. Gestational hypertension, early- and late-onset preeclampsia, severe preeclampsia, and composite HDP outcomes are frequently pooled despite their biological and prognostic differences. Moreover, HDP outcomes were secondary or exploratory endpoints in many metformin trials, limiting statistical power and increasing uncertainty around both favorable and neutral estimates [7,8,10].

The aim of this focused narrative review is therefore not to determine whether metformin functions as a universal preventive treatment for preeclampsia, but to critically evaluate whether specific metabolically enriched maternal phenotypes, treatment windows, comparators, or HDP subtypes may derive a secondary vascular benefit. The synthesis is structured around four interacting dimensions: maternal metabolic phenotype, timing of exposure relative to placentation, comparator and treatment response, and biological specificity of the hypertensive outcome. The review additionally examines direct and indirect mechanisms, placental transfer, fetal growth and long-term offspring safety, clinical positioning alongside established preventive interventions, and the postpartum cardiovascular and public health implications of metformin use.

## 2. Materials and Methods

### 2.1. Study Design and Review Objective

This article was designed as a focused narrative review of contemporary evidence rather than a systematic review or meta-analysis. A narrative approach was selected because the clinical question requires the integration of heterogeneous evidence, including randomized trials, observational cohorts, systematic reviews, pharmacokinetic studies, experimental placental research, biomarker studies, and long-term offspring follow-up. The objective was not to enumerate every publication containing the broad terms “metformin,” “pregnancy,” and “hypertension,” but to critically interpret the evidence directly relevant to whether metformin may modify gestational hypertension, preeclampsia, or composite hypertensive disorders of pregnancy in defined maternal phenotypes.

The review examined metformin exposure before or during pregnancy in relation to gestational hypertension, pregnancy-induced hypertension, preeclampsia, severe or early-onset preeclampsia, and composite HDP outcomes. Mechanistic, pharmacokinetic, placental, fetal growth, offspring, and postpartum cardiovascular studies were additionally included when they were necessary to interpret clinical findings. No pooled effect estimate was generated. No protocol was registered and no PRISMA flow diagram was produced because study selection was thematic, phenotype-based, and interpretative rather than exhaustive and systematic. The methodological structure and reporting were informed by principles used in the Scale for the Assessment of Narrative Review Articles (SANRA) [11].

### 2.2. Literature Sources and Search Strategy

Targeted searches were conducted in PubMed/MEDLINE, Scopus, Web of Science, and Google Scholar for publications from January 2015 through March 2026. Earlier landmark studies were retained when essential for the pathophysiology of HDP, placental angiogenic signaling, metformin pharmacology, or the clinical use of metformin in pregnancy. Search concepts combined ‘metformin’ with ‘pregnancy’, ‘gestational hypertension’, ‘pregnancy-induced hypertension’, ‘preeclampsia’, ‘severe preeclampsia’, ‘HELLP syndrome’, ‘gestational diabetes mellitus’, ‘type 2 diabetes mellitus’, ‘obesity’, ‘polycystic ovary syndrome’, ‘insulin resistance’, ‘endothelial dysfunction’, ‘oxidative stress’, ‘AMPK’, ‘placental dysfunction’, ‘angiogenic imbalance’, ‘sFlt-1’, ‘PlGF’, ‘fetal growth restriction’, and ‘small for gestational age’. Reference lists of key trials, systematic reviews, meta-analyses, and guidance documents were also screened.

The search process was iterative, as is appropriate for a focused narrative review. Initial broad searches were followed by phenotype-specific and mechanism-specific searches addressing GDM, T2DM, obesity, PCOS, placental dysfunction, angiogenic biomarkers, endothelial pathways, fetal growth, placental transfer, offspring outcomes, and postpartum cardiovascular risk. Reference lists of major randomized trials, systematic reviews, meta-analyses, and clinical guidance documents were examined to identify additional pivotal studies. Forward citation searching was also used to identify important updates and studies that challenged or refined earlier conclusions.

### 2.3. Search Terms

Search terms were combined using Boolean operators and included variations of: (“metformin” AND “pregnancy”); (“metformin” AND “preeclampsia”); (“metformin” AND “gestational hypertension”); (“metformin” AND “hypertensive disorders of pregnancy”); (“metformin” AND “gestational diabetes”); (“metformin” AND “type 2 diabetes” AND “pregnancy”); (“metformin” AND “obesity” AND “pregnancy”); (“metformin” AND “polycystic ovary syndrome” AND “pregnancy”); (“metformin” AND “placenta”); (“metformin” AND (“sFlt-1” OR “PlGF”)); (“metformin” AND “offspring”); and combinations involving endothelial dysfunction, AMPK, fetal growth, small for gestational age, aspirin, cardiovascular risk, and developmental programming. Search terms were adapted to the syntax of each database.

### 2.4. Eligibility Criteria

Eligible evidence included randomized controlled trials, prospective and retrospective cohort studies, systematic reviews, meta-analyses, clinical guidelines, pharmacokinetic studies, experimental human placental or vascular studies, and follow-up studies of metformin-exposed offspring. Studies were considered when they directly reported hypertensive outcomes or provided mechanistic, safety, fetal growth, or long-term evidence necessary for interpretation of the central clinical question.

Publications were excluded when metformin exposure could not be separated from other interventions, when pregnancy-specific outcomes were not reported, when hypertensive outcomes were not relevant to the review framework, or when the publication provided no additional clinical or mechanistic information beyond more complete reports. Case reports, conference abstracts without sufficient data, purely descriptive opinion pieces, and duplicate reports were generally excluded.

### 2.5. Evidence Selection and Prioritization

Because this was a focused narrative review, evidence was selected according to clinical relevance, methodological importance, and contribution to the predefined interpretative framework rather than through exhaustive inclusion of every search result. Priority was given to large randomized trials, contemporary systematic reviews and meta-analyses, population-based cohorts, major clinical guidelines, and studies directly reporting gestational hypertension, preeclampsia, or composite HDP outcomes. Smaller studies were retained when they addressed an otherwise underrepresented phenotype, clarified treatment timing or comparator effects, or provided important mechanistic or safety information.

Apparently discordant studies were intentionally retained when they differed in maternal phenotype, comparator treatment, metformin exposure, aspirin use, disease severity, or outcome definition, because explaining such heterogeneity was a central objective of the review.

### 2.6. Data Organization and Narrative Synthesis

Evidence was organized according to four predefined dimensions: maternal metabolic phenotype, timing of exposure relative to placentation, comparator and treatment response, and biological specificity of the hypertensive outcome. Within each phenotype, clinical findings were interpreted alongside study design, primary versus secondary outcome status, statistical power for HDP, supplemental insulin use, aspirin exposure, baseline vascular and renal disease, and fetal growth outcomes. The synthesis was qualitative and explanatory; no quantitative pooling or indirect comparison was undertaken.

### 2.7. Qualitative Critical Appraisal

Although no formal risk-of-bias instrument or certainty grading framework was applied, the evidentiary weight of the included studies was appraised qualitatively. Randomized trials were considered in relation to allocation methods, masking, attrition, treatment crossover, supplemental insulin use, outcome prespecification, and statistical power for HDP outcomes. Observational studies were evaluated with particular attention to confounding by indication, baseline renal and vascular disease, glycemic severity, aspirin exposure, comparator selection, adjustment strategy, and outcome ascertainment. Systematic reviews and meta-analyses were interpreted according to the methodological quality and clinical heterogeneity of their component studies. This approach supports critical interpretation but does not provide formal study-level risk-of-bias ratings, which remains a limitation of the focused review design. Nevertheless, the interpretation explicitly distinguishes randomized from observational evidence, primary from secondary outcomes, adequately powered from underpowered analyses, and experimental plausibility from demonstrated clinical effectiveness.

### 2.8. Data Extraction and Thematic Organization

For each publication, the following information was considered where available: study design, maternal population, baseline metabolic and cardiovascular phenotype, chronic hypertension or renal disease, previous HDP, gestational age at treatment initiation, metformin dose and duration, comparator, need for supplemental insulin, aspirin prophylaxis, glycemic and weight-related outcomes, hypertensive endpoint, fetal growth, neonatal outcomes, and placental or mechanistic findings. Evidence was organized into biological rationale, population-specific clinical findings, sources of heterogeneity, causal interpretation, clinical benefit–risk balance, and public health implications.

### 2.9. Hypertensive Outcome Framework and Qualitative Synthesis

Gestational hypertension, preeclampsia, preeclampsia with severe features, early- and late-onset disease, HELLP syndrome, and composite HDP outcomes were treated as related but biologically non-equivalent endpoints. A structured qualitative synthesis was used to identify convergent findings, explain discordant results, and distinguish effects on maternal metabolic or cardiovascular adaptation from effects on placenta-mediated disease. No quantitative pooling was performed because populations, treatment timing, comparator therapy, supplemental insulin use, aspirin exposure, disease severity, and outcome definitions differed substantially.

### 2.10. Clinical, Translational, and Public Health Perspective

The evidence was additionally interpreted from clinical, translational, and public health perspectives. The synthesis considered treatment feasibility, oral administration, cost, insulin access, monitoring requirements, placental transfer, fetal growth, and postpartum cardiovascular and metabolic risk. The objective was to support individualized counseling and precision prevention by identifying maternal phenotypes in which metabolic benefit might coexist with a secondary vascular advantage, while avoiding expansion of metformin use solely for an unproven HDP indication.

## 3. Biological and Pharmacological Rationale

HDP are systemic disorders in which maternal cardiovascular adaptation, placental function, metabolism, inflammation, and immunity interact. Abnormal placentation, placental malperfusion, oxidative stress, and angiogenic imbalance are central to many cases of preeclampsia, whereas maternal metabolic and cardiovascular vulnerability strongly influence the timing, severity, and clinical expression of disease [2,3]. The overlap with GDM, T2DM, obesity, and PCOS is therefore clinically meaningful rather than incidental [5,6].

Insulin resistance represents one of the principal links between metabolic disorders and hypertensive pregnancy complications. Physiological insulin resistance increases as pregnancy progresses, but excessive or pre-existing insulin resistance promotes compensatory hyperinsulinemia, dyslipidemia, adipose tissue inflammation, oxidative stress, and endothelial injury. These abnormalities are common in pregnancies complicated by obesity, GDM, T2DM, and PCOS and may increase susceptibility to gestational hypertension and preeclampsia [5,6,12].

Endothelial dysfunction is a defining component of the maternal syndrome of preeclampsia. Reduced nitric oxide bioavailability, increased vascular resistance, oxidative injury, inflammatory activation, and impaired vasodilatory responsiveness contribute to hypertension and maternal organ dysfunction [3,12]. Hyperglycemia and dyslipidemia may amplify reactive oxygen species generation, while obesity-related cytokines and adipokines further promote vascular activation.

The placenta provides the interface between maternal metabolic dysfunction and systemic vascular disease. In placenta-mediated preeclampsia, abnormal trophoblast invasion and impaired spiral artery remodeling contribute to placental hypoperfusion and the release of antiangiogenic mediators, particularly soluble fms-like tyrosine kinase-1 (sFlt-1) and soluble endoglin. Increased sFlt-1 reduces the bioavailability of vascular endothelial growth factor and placental growth factor (PlGF), thereby promoting systemic endothelial dysfunction [3]. The sFlt-1/PlGF ratio is consequently useful for characterizing angiogenic imbalance and identifying pregnancies with a predominantly placenta-mediated phenotype, although it should be interpreted alongside gestational age and clinical findings [13,14].

Metformin may influence HDP through two conceptually distinct routes. Indirect effects arise from improvements in maternal glycemia, insulin sensitivity, gestational weight gain, lipid metabolism, and systemic inflammatory burden. These effects may reduce maternal metabolic and endothelial stress without necessarily modifying the initiating placental lesion. Therefore, improved glycemic control or lower gestational weight gain cannot automatically be interpreted as prevention of placenta-mediated preeclampsia [7,8,12,13,15].

Potential direct vascular and placental actions are biologically plausible but are supported mainly by experimental and biomarker evidence. Metformin inhibits mitochondrial respiratory chain complex I, alters cellular energy status, and activates AMP-activated protein kinase (AMPK). Downstream AMPK signaling may enhance endothelial nitric oxide synthase activity and nitric oxide bioavailability, improve vascular responsiveness, reduce reactive oxygen species, and inhibit pro-inflammatory NF-κB signaling [12]. Experimental trophoblast findings are dose-dependent: supratherapeutic metformin concentrations impaired oxidative metabolism and differentiation, whereas concentrations in the therapeutic range did not strongly affect these processes [15].

Experimental studies using primary human placental and vascular tissues provide a more specific angiogenic rationale. Brownfoot et al. demonstrated that metformin reduced sFlt-1 and soluble endoglin secretion from endothelial cells, cytotrophoblasts, and preeclamptic placental explants. Metformin also reduced markers of endothelial dysfunction, restored impaired vasodilation in maternal vessels, and improved angiogenic sprouting in ex vivo vascular preparations [12]. A subsequent systematic review of placental disease biomarkers found that metformin exposure was associated with lower C-reactive protein and inflammatory cytokines and reduced release of sFlt-1 and soluble endoglin in experimental placental and vascular studies [13].

These findings establish mechanistic plausibility but should not be equated with demonstrated clinical effectiveness. Experimental concentrations, tissue models, treatment duration, and underlying placental pathology may differ substantially from clinical exposure. Furthermore, favorable effects on isolated biomarkers do not necessarily translate into lower rates of gestational hypertension, preeclampsia, or maternal organ complications [7,8,9,10,12,13,15].

The sFlt-1/PlGF ratio may nevertheless serve as a candidate effect modifier and stratification biomarker in future metformin trials. A markedly abnormal ratio is associated with angiogenic imbalance, placenta-mediated disease, greater disease severity, and an increased risk of adverse maternal and perinatal outcomes [14]. It may therefore identify a phenotype that is biologically less likely to respond to a primarily metabolic intervention initiated after placentation, although this hypothesis has not yet been tested prospectively. Conversely, women with a less pronounced angiogenic disturbance but substantial insulin resistance, obesity-related inflammation, or metabolic endothelial dysfunction may represent a biologically different subgroup. This distinction may be particularly relevant in women with GDM, in whom the sFlt-1/PlGF ratio retains predictive value, although the underlying metabolic disorder may influence angiogenic biomarker concentrations [16]. The ratio should not currently be regarded as a validated test for selecting women for metformin treatment; rather, its prospective incorporation could clarify whether treatment effects differ across placental and metabolic phenotypes [14,16].

Timing is likely to be important. In most GDM trials, metformin is initiated after diagnosis in the late second trimester, when trophoblast invasion and early spiral artery remodeling have largely occurred [3,7,10]. Treatment at this stage may improve maternal glycemia, insulin sensitivity, and gestational weight gain but is unlikely to reverse established placental maldevelopment. Earlier exposure in women with T2DM or PCOS could theoretically influence placental adaptation, yet clinical evidence remains inconsistent [8,9]. Dose, adherence, maternal renal function, baseline cardiovascular disease, aspirin prophylaxis, comparator treatment, and the need for supplemental insulin may further modify any apparent effect. Earlier initiation also increases the duration of direct fetal exposure.

A final consideration is placental transfer. Unlike insulin, metformin readily crosses the human placenta, and fetal or umbilical cord concentrations in late pregnancy may approach those measured in the maternal circulation [17]. Studies using matched maternal plasma, fetal plasma, and placental tissue have similarly demonstrated close correlations between maternal and fetal metformin concentrations and confirmed direct placental and fetal exposure [18]. Such exposure does not preclude treatment for established metabolic indications, but it broadens the benefit–risk assessment beyond short-term glycemic control. Metformin may influence placental mitochondrial respiration, cellular ATP production, oxidative stress pathways, AMPK–mTOR nutrient sensing, fetal growth, and postnatal body composition trajectories [18].

Available evidence is broadly reassuring regarding congenital anomalies and short-term neonatal safety. However, meta-analytic evidence indicates that metformin-exposed neonates may have lower birthweight, lower ponderal index, and differences in selected anthropometric measurements compared with insulin-exposed neonates, even when maternal glycemic control is broadly comparable [19]. These findings may reflect reduced pathological overgrowth, direct placental or fetal effects, or differences in the maternal populations receiving each treatment.

Long-term findings remain heterogeneous. A large register-based cohort found no increased risk of childhood obesity, diabetes, hypertension, or motor–social developmental difficulties after metformin exposure compared with insulin, although an increased risk of small-for-gestational-age birth was observed [20]. A systematic review and meta-analysis also found no association between intrauterine metformin exposure and adverse neurodevelopmental outcomes through childhood, including follow-up extending to 14 years in some studies [21]. Nevertheless, evidence concerning offspring blood pressure, vascular function, body composition, glucose and lipid metabolism, puberty, and adult cardiovascular health remains limited. These uncertainties do not demonstrate long-term harm but support indication-specific use and prolonged follow-up rather than expansion of fetal exposure solely for an unproven HDP prevention indication [18,19,20,21].

The clinical phenotype of HDP also matters. Early-onset preeclampsia is more strongly associated with placental malperfusion and profound angiogenic imbalance, whereas late-onset disease often reflects a broader interaction between placental demand and maternal cardiovascular or metabolic vulnerability [2,3]. Metformin might therefore be more likely to influence late-onset or metabolically enriched disease than severe early placenta-mediated preeclampsia, but this remains a hypothesis because existing studies rarely report outcomes by gestational age at onset, angiogenic phenotype, or disease severity.

Metformin should also be distinguished from established preventive interventions. Low-dose aspirin targets platelet and placental pathways in women at increased risk of preeclampsia, while optimization of chronic hypertension, diabetes, and renal disease reduces preventable maternal risk [1,2]. Any investigation of metformin should therefore evaluate it as an adjunct to, rather than a substitute for, established risk assessment, aspirin prophylaxis, blood pressure surveillance, and maternal–fetal monitoring. The principal biological pathways linking metformin with hypertensive disorders of pregnancy are summarized in Table 1.

## 4. Clinical Evidence by Maternal Population

### 4.1. Gestational Diabetes Mellitus

GDM is the most extensively studied setting for metformin use during pregnancy. Compared with insulin, metformin is orally administered, is generally associated with less gestational weight gain and fewer maternal hypoglycemic episodes, and may reduce the treatment burden for women who prefer to avoid injections. However, a clinically important proportion of women require supplemental insulin, indicating that GDM encompasses metabolically heterogeneous phenotypes with different degrees of β-cell dysfunction and insulin resistance [8,22,23].

Evidence regarding hypertensive outcomes is inconsistent. In the meta-analysis by Kalafat et al., metformin was associated with a lower risk of pregnancy-induced hypertension compared with insulin, whereas the reduction in preeclampsia was not statistically significant [7]. Conversely, a later meta-analysis of 24 randomized trials involving 4934 women reported a lower risk of preeclampsia with metformin than with insulin, but no significant reduction in gestational hypertension [23]. These discordant findings illustrate the importance of separating gestational hypertension from preeclampsia rather than treating them as interchangeable manifestations of a single outcome.

A broader meta-analysis across maternal indications found that metformin reduced gestational weight gain and was associated with a modest reduction in preeclampsia, but considerable clinical heterogeneity and an increased frequency of gastrointestinal adverse effects limited the certainty and generalizability of this estimate [8]. The favorable pooled effects may partly reflect differences between metformin and insulin in maternal weight gain, hypoglycemia, baseline metabolic severity, and the requirement for treatment intensification rather than a direct anti-preeclamptic action.

Contemporary randomized trials provide a more cautious perspective. In the EMERGE trial, earlier addition of metformin to usual GDM care did not significantly improve the primary composite of insulin initiation or persistent fasting hyperglycemia, although metformin was associated with delayed insulin initiation, less gestational weight gain, and smaller neonatal size [22]. Hypertensive outcomes were not the primary basis on which the trial was designed or powered, limiting conclusions regarding HDP prevention.

Similarly, the SUGAR-DIP trial did not demonstrate noninferiority of a sequential oral glucose-lowering strategy initiated with metformin compared with insulin for the primary large-for-gestational-age outcome. Pregnancy-induced hypertension, preeclampsia, and most other secondary outcomes did not differ materially between treatment groups [10]. The trial therefore challenges the assumption that metabolic advantages or avoidance of insulin necessarily translate into a lower incidence of HDP.

Population-based findings are also largely neutral. In the Scotland–Sweden analysis, metformin exposure among women with diabetes was not associated with altered risks of composite HDP or preeclampsia after adjustment for measured confounders [9]. Real-world comparisons remain vulnerable to residual confounding because women selected for metformin may differ from insulin-treated women in baseline glycemic severity, body mass index, renal function, treatment adherence, and clinician prescribing preferences.

The most defensible interpretation is therefore that metformin may reduce selected metabolic contributors to hypertensive risk in some women with GDM, particularly excessive gestational weight gain and insulin resistance, but it has not been established as an HDP-preventive treatment. Most GDM trials were primarily designed for glycemic, neonatal, or composite outcomes and included relatively few hypertensive events. Consequently, both favorable and neutral HDP estimates should be regarded as exploratory unless confirmed in trials with prespecified, adequately powered, phenotype-specific hypertensive endpoints.

Future GDM studies should separately report gestational hypertension, preeclampsia, preeclampsia with severe features, medically indicated preterm delivery, and early- versus late-onset disease. They should additionally account for gestational age at treatment initiation, metformin dose, adherence, supplemental insulin, aspirin prophylaxis, baseline body mass index, and glycemic severity. Analyses of metformin-only responders and women requiring supplemental insulin may help identify whether treatment response marks a clinically relevant metabolic phenotype.

### 4.2. Pregestational Type 2 Diabetes Mellitus

Women with pregestational T2DM have a high baseline risk of HDP because insulin resistance frequently coexists with obesity, chronic hypertension, renal disease, dyslipidemia, and established vascular dysfunction. In this population, metformin may be continued from before conception or added to insulin during pregnancy, making its independent effect on hypertensive outcomes difficult to isolate.

The MiTy randomized trial evaluated metformin added to insulin in women with T2DM. Metformin improved glycemic control, reduced insulin requirements and gestational weight gain, and reduced several measures of neonatal size and adiposity. However, hypertensive disorders occurred in 23% of both treatment groups, providing no evidence of an HDP benefit [24]. The trial also identified a higher proportion of small-for-gestational-age infants in the metformin group, emphasizing that reductions in average birthweight or fetal overgrowth are not uniformly favorable in women with potentially impaired placental reserve.

The MOMPOD trial similarly evaluated metformin added to insulin in women with preexisting T2DM or diabetes diagnosed before 23 weeks. Metformin did not significantly reduce the composite adverse neonatal outcome, although the odds of a large-for-gestational-age infant were lower [25]. A planned secondary analysis specifically examining preterm preeclampsia found no reduction in preeclampsia requiring delivery before either 37 or 34 weeks and no significant differences in measured cardiovascular, inflammatory, or angiogenic biomarkers [26]. This analysis is particularly relevant because it evaluated a more biologically specific and clinically severe hypertensive phenotype than composite HDP alone.

Observational evidence is compatible with these neutral randomized findings. A target trial emulation comparing continuation with discontinuation of metformin in women using metformin plus insulin at conception found similar risks for most maternal and neonatal outcomes, including gestational hypertension and preeclampsia. A possible increase in SGA was observed in one database and requires further evaluation [27]. The Scotland–Sweden cohort likewise found no adjusted association between metformin use and either composite HDP or preeclampsia [9].

These findings indicate that the metabolic advantages of metformin in T2DM—lower insulin requirements, less gestational weight gain, and fewer excessively large infants—do not consistently translate into reduced hypertensive disease. Baseline nephropathy, chronic hypertension, vascular complications, diabetes duration, aspirin use, and severity of hyperglycemia may dominate HDP risk and may also influence whether metformin is continued or discontinued.

Residual confounding remains particularly important in nonrandomized comparisons. Women who continue metformin may have better renal function, less advanced vascular disease, lower insulin requirements, or better treatment adherence than women managed predominantly with insulin. Conversely, combined metformin and insulin therapy may identify women with more severe metabolic disease. Even extensive statistical adjustment may therefore fail to distinguish the drug effect from the clinical reasons for prescribing it.

Metformin may remain useful in selected women with T2DM for metabolic optimization, but current evidence does not support its addition or continuation primarily for preventing HDP. In women with chronic hypertension, nephropathy, fetal growth restriction, or suspected placental insufficiency, fetal growth velocity and placental function should be monitored rather than assuming that lower fetal size represents a beneficial treatment effect.

### 4.3. Obesity Without Diabetes

Obesity increases the risk of HDP through insulin resistance, adipose tissue inflammation, oxidative stress, endothelial dysfunction, altered cardiovascular adaptation, and placental dysfunction. These mechanisms created a rationale for evaluating metformin in pregnant women with overweight or obesity but without diabetes.

The GRoW randomized trial evaluated metformin in addition to structured dietary and lifestyle advice in women with overweight or obesity. Metformin modestly reduced weekly gestational weight gain but did not reduce the primary outcome of birthweight above 4000 g and did not improve overall pregnancy or birth outcomes [28]. Thus, a measurable metabolic or weight-related effect did not translate into broad obstetric benefit.

The EMPOWaR randomized trial similarly tested metformin in obese women with normal glucose tolerance. Metformin did not improve the primary standardized birthweight outcome or provide convincing evidence of improved maternal or fetal outcomes, leading the investigators to conclude that metformin should not be used to improve pregnancy outcomes in obese women without diabetes [29].

Meta-analytic findings are less consistent. Kalafat et al. found a nonsignificant reduction in preeclampsia among obese women receiving metformin compared with placebo, with substantial statistical heterogeneity and wide confidence intervals [7]. Broader analyses have suggested lower gestational weight gain and possibly lower preeclampsia rates across mixed maternal indications, but these estimates combine populations with different baseline metabolic risks and comparators [8].

The neutral findings of GRoW and EMPOWaR are therefore more clinically persuasive than isolated favorable secondary or pooled estimates. Obesity alone is not an established indication for metformin in pregnancy, and current evidence does not support routine fetal exposure solely to reduce gestational weight gain or prevent HDP. Lifestyle support, appropriate gestational weight-gain counseling, early diabetes screening, standard preeclampsia risk assessment, aspirin when indicated, and blood pressure surveillance remain the preferred strategies.

Future trials should not treat all women with obesity as one phenotype. Insulin resistance, metabolic syndrome, chronic hypertension, inflammatory biomarkers, fat distribution, previous GDM, and prior HDP may identify subgroups with substantially different risks and potential treatment responses. However, such phenotype-specific benefit has not yet been demonstrated.

### 4.4. Polycystic Ovary Syndrome

PCOS is a heterogeneous reproductive and metabolic disorder characterized by variable combinations of hyperandrogenism, ovulatory dysfunction, obesity, insulin resistance, and chronic low-grade inflammation. Women may begin metformin before conception for metabolic or reproductive indications and continue it during pregnancy, resulting in an earlier and longer exposure window than is typical in GDM.

The PregMet2 randomized, double-blind, placebo-controlled trial evaluated metformin from the late first trimester until delivery. The primary composite of late miscarriage and preterm birth was numerically lower with metformin but did not reach conventional statistical significance in the individual trial. A pooled individual participant analysis incorporating two earlier trials suggested a reduction in late miscarriage or preterm birth. However, PregMet2 found no significant benefit for its secondary outcomes, which included preeclampsia and pregnancy-induced hypertension [30]. Importantly, the study was designed primarily around late miscarriage and preterm birth rather than HDP and was not powered to establish a definitive effect on hypertensive outcomes.

Evidence from meta-analyses depends strongly on study selection. Analyses combining randomized and observational studies have sometimes reported reductions in pregnancy-induced hypertension or preeclampsia, but they are vulnerable to confounding, variable PCOS diagnostic criteria, differing treatment durations, and inconsistent outcome definitions. By contrast, a meta-analysis restricted to randomized controlled trials found no significant reduction in preeclampsia, although metformin was associated with a lower risk of preterm delivery [31].

This distinction is important because women prescribed metformin may differ from untreated women in body mass index, insulin resistance, infertility treatment, androgen excess, prior pregnancy loss, and clinician assessment of metabolic risk. Observational associations may therefore overestimate benefit if treatment selection is linked to characteristics that also influence pregnancy outcomes.

The available evidence supports neither routine continuation nor routine discontinuation of metformin solely on the basis of a PCOS diagnosis. Decisions should be based on the original metabolic or reproductive indication, maternal glycemic status, body mass index, tolerability, fetal growth, and the balance between maternal benefit and duration of fetal exposure. Current evidence does not justify continuing metformin throughout pregnancy specifically to prevent HDP.

Future PCOS trials should stratify participants according to obesity, biochemical insulin resistance, hyperandrogenism, fertility treatment, prior HDP, and metformin exposure before conception. Adequately powered HDP endpoints and longer-term offspring follow-up are necessary to determine whether any benefit is confined to metabolically enriched PCOS phenotypes.

### 4.5. Integrated Clinical Interpretation

Across maternal populations, evidence is considerably more consistent for metabolic effects than for hypertensive outcomes. Metformin often reduces gestational weight gain, insulin requirements, maternal hypoglycemia, and excessive fetal growth, but these effects do not reliably produce lower rates of gestational hypertension or preeclampsia [8,9,10,22,23,24,25,26,27,28,29,30,31].

The apparent treatment effect depends on maternal phenotype and comparator. In GDM, comparisons with insulin may partly reflect differences in weight gain, hypoglycemia, and baseline disease severity. In T2DM, chronic hypertension, nephropathy, and vascular disease may dominate risk and create substantial confounding by indication. In obesity without diabetes, two large placebo-controlled trials did not establish clinically meaningful pregnancy benefit. In PCOS, favorable pooled findings are weakened when attention is restricted to randomized evidence.

Timing may also influence the apparent effect. Metformin is generally initiated after mid-pregnancy in GDM, when early placental remodeling has already occurred, whereas exposure may begin before conception or during the first trimester in T2DM and PCOS. Nevertheless, earlier exposure has not been shown consistently to prevent HDP and also increases the duration of fetal exposure.

Outcome specificity is equally important. A reduction in pregnancy-induced hypertension cannot automatically be extrapolated to preeclampsia, and neutral composite HDP findings may conceal different effects on gestational hypertension, late-onset preeclampsia, or severe early placenta-mediated disease. Most available studies do not report HDP according to gestational age at onset, severity, angiogenic phenotype, or medically indicated preterm delivery.

Current evidence therefore supports metformin as an indication-based metabolic therapy with a possible but unproven secondary vascular benefit in selected phenotypes. It should not be prescribed as a universal preventive treatment for HDP and should not replace aspirin prophylaxis when indicated, blood pressure surveillance, optimization of chronic hypertension and diabetes, renal assessment, or maternal–fetal monitoring. The clinical evidence on metformin and hypertensive outcomes across different maternal populations is summarized in Table 2.

## 5. Discussion

### 5.1. Overall Interpretation of the Evidence

The central finding of this review is a persistent gap between mechanistic plausibility and demonstrated clinical effectiveness. Metformin influences insulin resistance, gestational weight gain, inflammatory signaling, oxidative stress, mitochondrial activity, endothelial function, and experimental placental angiogenic pathways relevant to HDP [12,13,15]. Nevertheless, clinical studies do not demonstrate a uniform reduction in gestational hypertension, preeclampsia, or composite HDP across maternal populations [7,8,9,10,22,23,24,25,26,27,28,29,30,31].

The most defensible interpretation is that metformin may function as a metabolic and vascular modifier rather than as a specific anti-preeclamptic therapy. Any hypertensive benefit is likely to be indirect, modest, and dependent on maternal phenotype, treatment timing, comparator therapy, and the biological subtype of HDP. This interpretation is consistent with contemporary reviews emphasizing that metformin’s established pregnancy benefits relate principally to glycemic control, gestational weight gain, insulin requirements, and excessive fetal growth, whereas its effects on preeclampsia remain uncertain [32].

The distinctive contribution of this review is a four-dimensional interpretative framework integrating maternal metabolic phenotype, timing of exposure relative to placentation, comparator and treatment response, and biological specificity of the hypertensive outcome. This framework helps explain why some pooled analyses suggest benefit while contemporary randomized and population-based studies remain neutral. It also shifts the clinical question from whether metformin prevents preeclampsia in general to whether particular metabolically enriched phenotypes may derive a secondary vascular benefit without compromising placental reserve or fetal growth.

Positive pooled estimates should be interpreted alongside neutral contemporary evidence. Meta-analyses have reported reductions in pregnancy-induced hypertension or preeclampsia in some comparisons, particularly when metformin was compared with insulin [7,8,23]. However, hypertensive outcomes were usually secondary endpoints, event numbers were often limited, outcome definitions differed, and pooled estimates combined clinically heterogeneous maternal populations. In contrast, SUGAR-DIP, MiTy, MOMPOD, PregMet2, GRoW, EMPOWaR, and large adjusted cohort analyses did not establish a consistent hypertensive benefit [9,10,24,25,26,27,28,29,30].

These differences do not merely represent statistical disagreement. They reflect different clinical questions. Comparisons of metformin with insulin evaluate both pharmacological and treatment-related differences, including gestational weight gain, hypoglycemia, injection burden, and the metabolic severity prompting insulin therapy. Placebo-controlled trials examine whether metformin provides benefit beyond usual care, whereas observational studies are influenced by the clinical characteristics that determine treatment selection.

Metformin’s most reproducible pregnancy effects remain metabolic rather than hypertensive. Lower gestational weight gain, reduced insulin requirements, improved glycemic control, and fewer excessively large neonates may improve the overall course of selected pregnancies [8,22,23,24,25]. However, these effects cannot automatically be interpreted as prevention of placenta-mediated disease, particularly when preeclampsia is not a prespecified or adequately powered primary outcome.

### 5.2. Disease Phenotype, Timing, and Biological Specificity

HDP should not be regarded as a single biological entity. Gestational hypertension, late-onset preeclampsia, early-onset preeclampsia, preeclampsia with severe features, eclampsia, and HELLP syndrome differ in the relative contributions of maternal cardiovascular vulnerability, metabolic dysfunction, placental malperfusion, inflammation, and angiogenic imbalance [2,3]. Pooling these outcomes may increase statistical power but reduces biological and clinical interpretability.

A therapy that improves insulin sensitivity and limits gestational weight gain may plausibly influence metabolically enriched gestational hypertension or late-onset preeclampsia more than severe early placenta-mediated disease. This possibility is supported conceptually by the distinction between maternal metabolic–endothelial stress and profound placental angiogenic dysfunction, but it has not been demonstrated prospectively. Most metformin studies did not distinguish HDP according to gestational age at onset, severity, or angiogenic phenotype [7,8,9,10,22,23,24,25,26,27,28,29,30,31].

Timing provides a further explanation for heterogeneous findings. In most GDM trials, metformin is started after diagnosis in the late second trimester, when trophoblast invasion and early spiral artery remodeling have largely occurred. Treatment at this stage may improve glycemia, insulin sensitivity, and gestational weight gain but is unlikely to reverse established placental maldevelopment [3,7,10,22].

In T2DM and PCOS, metformin exposure may begin before conception or during the first trimester. Earlier exposure could theoretically influence maternal metabolic adaptation and early placental development, but clinical evidence does not demonstrate consistent prevention of HDP in these populations [24,25,26,27,30,31]. Earlier initiation also increases the duration of placental and fetal exposure and therefore cannot be assumed to provide a more favorable benefit–risk balance.

The sFlt-1/PlGF ratio may provide a useful framework for future phenotype-based trials. A markedly abnormal ratio reflects angiogenic imbalance and a predominantly placenta-mediated process, whereas a less disturbed angiogenic profile in a woman with substantial obesity, insulin resistance, or metabolic inflammation may identify a different disease pathway [14,16]. However, the ratio is not currently validated for selecting women for metformin treatment.

Future trials should prespecify gestational hypertension, preeclampsia, severe preeclampsia, and medically indicated preterm delivery as separate outcomes. Preeclampsia should additionally be classified according to onset before 34 weeks, before 37 weeks, and at term. Integration of sFlt-1, PlGF, the sFlt-1/PlGF ratio, maternal cardiovascular measures, and metabolic biomarkers may determine whether metformin effects vary between placental and metabolically enriched phenotypes.

### 5.3. Comparator Effects, Treatment Response, and Causal Interpretation

The choice of comparator strongly influences the apparent effect of metformin. Comparisons with insulin capture not only differences between two pharmacological agents but also differences in route of administration, maternal weight gain, risk of hypoglycemia, treatment burden, and the metabolic severity that prompted insulin initiation [7,8,23,32]. Insulin-treated groups may include a greater proportion of women with higher fasting glucose, more pronounced β-cell dysfunction, earlier pharmacological treatment, or failure of previous oral therapy. Consequently, an apparent advantage of metformin over insulin cannot automatically be interpreted as an independent antihypertensive or placental effect. Comparisons with placebo, lifestyle intervention, usual care, or treatment discontinuation address different causal questions and should not be regarded as interchangeable evidence [9,10,22,24,25,26,27,28,29].

Supplemental insulin is another important source of clinical and analytical heterogeneity. A substantial proportion of women allocated to metformin in GDM trials require additional insulin to achieve glycemic targets [10,22,33,34,35]. In a multicenter cohort of 2891 women initially treated with metformin, 23.7% required combined therapy with insulin; higher pregestational body mass index, higher fasting glucose on the diagnostic oral glucose tolerance test, and earlier gestational age at metformin initiation independently predicted metformin monotherapy failure [33]. These findings support the interpretation that the need for supplemental insulin reflects a more severe metabolic phenotype rather than simply inadequate adherence or pharmacological failure.

GDM itself is heterogeneous, and standardized treatment comparisons may obscure clinically relevant subgroups. A systematic review of precision treatment identified previous GDM, maternal body mass index, and glucose concentrations at diagnosis as potential markers of the need for pharmacological escalation [34]. Women who achieve glycemic control with metformin alone may therefore have less β-cell dysfunction, lower fasting hyperglycemia, or a more insulin-resistant but insulin-responsive phenotype than women requiring combined metformin and insulin therapy [33,34]. Treatment response may consequently operate as a marker of baseline disease severity and underlying pathophysiology, rather than solely as a consequence of drug effectiveness.

Analyses based only on randomized treatment assignment may obscure responder and non-responder phenotypes because women who subsequently require supplemental insulin remain within the metformin allocation group. However, per-protocol, as-treated, or treatment response analyses can introduce selection bias because post-randomization treatment escalation is associated with baseline glycemia, body mass index, gestational age at diagnosis, adherence, gastrointestinal tolerability, and clinician management [22,33,34]. Future studies should therefore retain intention-to-treat analyses as the primary approach while also reporting prespecified, carefully adjusted exploratory analyses according to supplemental insulin requirement, time to treatment escalation, and achieved glycemic control.

The wider diabetes-in-pregnancy literature also demonstrates substantial inconsistency in study design and outcome reporting. A systematic review of 108 clinical trials and observational studies found marked variation in treatment arms, study populations, glycemic measures, and maternal and fetal outcomes, with no uniform set of outcome metrics [35]. Such heterogeneity limits direct comparison between trials and may contribute to apparently conflicting estimates of gestational hypertension and preeclampsia. Standardized reporting is therefore necessary not only for glycemic outcomes but also for HDP definitions, gestational age at diagnosis, severity, treatment escalation, and maternal and neonatal consequences.

Observational studies are particularly vulnerable to confounding by indication. Continuation of metformin in women with T2DM may depend on renal function, glycemic control, body mass index, diabetes duration, clinician preference, previous response to therapy, and the presence of chronic hypertension or vascular complications [9,27]. Women who continue metformin may therefore have better renal function, less advanced vascular disease, lower overall metabolic severity, or better adherence than women managed predominantly with insulin. Conversely, combined metformin and insulin therapy may identify women with more severe diabetes, higher insulin resistance, or failure of metformin monotherapy [24,27,33].

The target trial emulation by Yland et al. attempted to reduce this bias by comparing continuation with discontinuation of metformin among women already treated with metformin and insulin at conception [27]. Nevertheless, even carefully designed observational analyses cannot fully account for unmeasured determinants of prescribing, including clinician concern regarding renal or placental function, previous treatment tolerance, socioeconomic barriers, or subtle differences in diabetes severity. Similarly, the neutral Scotland–Sweden findings after inverse probability weighting do not exclude either a small treatment effect or residual confounding [9].

Even sophisticated adjustment cannot eliminate residual confounding when the treatment decision is closely related to the severity and prognosis of the underlying disease. A favorable association in a smaller or less adjusted cohort and a neutral association in a larger weighted analysis may therefore both be internally valid while representing different clinical populations. In practical terms, an observed association may partly reflect which women were selected to receive or continue metformin rather than the pharmacological effect of metformin itself [9,27,33,34].

Aspirin co-administration requires similarly explicit consideration. Low-dose aspirin is an established preventive intervention in women at increased risk of preeclampsia and may reduce the absolute event rate against which any additional metformin effect is assessed [1,2,36]. Meta-analytic evidence indicates that aspirin effectiveness varies according to gestational age at initiation and dose, with greater benefit generally observed when prophylaxis is started by 16 weeks [36]. Therefore, imbalance between treatment groups in aspirin eligibility, dose, timing, adherence, or discontinuation could either exaggerate or obscure an apparent association between metformin and HDP.

Future metformin trials should report the proportion of women eligible for aspirin, the risk assessment method used, aspirin dose, gestational age at initiation, adherence, and treatment discontinuation. Analyses should adjust or stratify for aspirin exposure and, where sample size permits, evaluate a prespecified metformin-by-aspirin interaction. Without such information, a neutral metformin result may reflect effective background prophylaxis, whereas an apparently favorable effect may reflect lower or delayed aspirin use in the comparator group.

Outcome classification is another major source of potential bias. Gestational hypertension, preeclampsia without severe features, preeclampsia with severe features, eclampsia, and HELLP syndrome differ substantially in clinical severity and associated cardiovascular morbidity [2,3,37]. In a population-based analysis of more than 15 million deliveries, cardiovascular severe maternal morbidity increased progressively across HDP subtypes and was particularly high in HELLP syndrome and severe preeclampsia [37]. These outcomes should therefore not be treated as clinically equivalent components of a single composite endpoint.

Combining biologically and prognostically distinct diagnoses may increase the number of events and statistical power but can conceal opposing or phenotype-specific treatment effects. A reduction in gestational hypertension, for example, does not establish prevention of severe or preterm preeclampsia. Future studies should report chronic hypertension with superimposed preeclampsia, gestational hypertension, preeclampsia with and without severe features, eclampsia, and HELLP syndrome separately. They should additionally record gestational age at diagnosis and delivery, maternal organ dysfunction, antihypertensive treatment, magnesium sulfate use, medically indicated preterm delivery, fetal growth abnormalities, neonatal intensive care admission, and perinatal mortality [2,3,35,37].

### 5.4. Clinical Benefit-Risk Balance

The absence of proven HDP prevention does not negate the clinical value of metformin for established metabolic indications. In appropriately selected women with GDM or T2DM, metformin may provide clinically meaningful benefits, including oral administration, lower gestational weight gain, reduced insulin requirements, fewer maternal hypoglycemic episodes, and a lower frequency of excessive fetal growth or neonatal hypoglycemia in some comparisons with insulin [8,22,23,24,25,32]. These benefits may improve treatment acceptability and access, particularly when insulin administration creates substantial financial, logistical, or psychological barriers.

However, maternal metabolic benefit should not be conflated with prevention of placenta-mediated disease. Improvements in glycemia, insulin requirements, or gestational weight gain do not consistently translate into lower rates of gestational hypertension, preeclampsia, or preterm preeclampsia [7,8,9,10,23,24,25,26,27]. Metformin should therefore be prescribed according to an established metabolic indication rather than primarily to prevent HDP.

Metformin should also not delay the initiation or intensification of insulin when glycemic targets are not achieved. A clinically relevant proportion of women initially managed with metformin require supplemental insulin, particularly those with higher fasting glucose, greater pregestational body mass index, or earlier diagnosis and treatment initiation [22,33,34]. Treatment escalation should be interpreted as appropriate phenotype-responsive care rather than as a therapeutic failure.

The maternal benefit–risk balance must be considered alongside placental reserve and fetal growth. Reducing pathological fetal overgrowth may be advantageous in women with marked hyperglycemia and a high risk of an LGA or macrosomic infant [23,24,25,38]. Conversely, lower birthweight or SGA may be undesirable in pregnancies complicated by chronic hypertension, nephropathy, maternal vascular disease, fetal growth restriction, or suspected placental insufficiency [20,24,27,39].

In the MiTy trial, metformin added to insulin reduced maternal weight gain, insulin requirements, and measures of neonatal size but increased the proportion of SGA infants [24]. A subsequent MiTy analysis found that both metformin exposure and the presence of chronic hypertension or nephropathy independently predicted SGA, with the highest absolute risk observed among metformin-treated women with these vascular or renal comorbidities [39]. These findings support a cautious and individualized approach in women with T2DM and reduced placental or maternal vascular reserve.

Nevertheless, the SGA signal is not uniform across maternal populations or comparators. Register-based analyses have found an increased SGA risk when metformin was compared with insulin, but not consistently when it was compared with non-pharmacological GDM management [20,27]. Similarly, recent GDM evidence suggests that metformin is associated with lower average birthweight but does not consistently increase SGA [22,38]. The apparent growth effect therefore depends on maternal indication, baseline hyperglycemia, comparator treatment, placental function, and the clinical reasons for treatment selection.

Mean birthweight alone is insufficient for evaluating fetal safety. A lower mean may reflect a desirable reduction in pathological overgrowth, a shift toward appropriate-for-gestational-age birthweight, or an unfavorable increase in growth restriction. Trials should therefore report the complete birthweight distribution, LGA, SGA, severe SGA, fetal growth velocity, gestational age at delivery, and the indication for preterm delivery rather than interpreting lower neonatal size as intrinsically beneficial or harmful [19,20,24,38,39].

Serial fetal growth assessment may be particularly appropriate in women with T2DM, chronic hypertension, nephropathy, vascular disease, abnormal uterine or umbilical artery Doppler findings, or emerging evidence of placental insufficiency. In these settings, continuation of metformin should be reviewed together with glycemic control, maternal insulin requirements, fetal growth trajectory, and the overall clinical indication. Current evidence does not establish that discontinuation improves fetal growth, but it supports heightened caution where fetal undernutrition is already plausible [24,27,32,39].

Direct placental and fetal exposure further distinguishes metformin from insulin. Because fetal concentrations may approach maternal concentrations, the benefit–risk assessment extends beyond maternal glycemic control to potential effects on placental mitochondrial function, fetal growth, and developmental programming [14,16,17,18,19]. Short-term evidence is generally reassuring regarding major congenital anomalies, neonatal morbidity, and neurodevelopment [18,19,20,21,38,40,41].

Long-term evidence is increasingly reassuring but remains incomplete. A systematic review and meta-analysis evaluating children aged approximately 5–11 years found no clear adverse differences in body mass index, body composition, metabolic markers, obesity, diabetes, or motor–social development after metformin exposure compared with insulin [40]. A more recent meta-analysis including 7975 metformin-exposed children found broadly comparable adiposity measures in later childhood, although a slightly higher body mass index was observed at ages 1–3 years and findings varied according to maternal indication [41].

These reassuring findings should be interpreted cautiously because certainty remains limited by attrition, heterogeneous maternal indications, differences in comparator groups, variable metformin dose and duration, and relatively short follow-up compared with the life course over which cardiometabolic disease develops [19,20,21,32,38,40,41]. Current evidence does not demonstrate clinically important long-term harm, but neither does it exclude smaller effects on growth trajectory, puberty, vascular function, glucose metabolism, or adult cardiovascular risk.

The appropriate clinical position is therefore neither unrestricted use nor categorical avoidance. Metformin should be used when the expected maternal metabolic benefit is clinically meaningful, glycemic control is achievable, and there is no emerging concern regarding fetal growth or placental insufficiency. Its use solely to prevent HDP is not currently justified.

Metformin should remain an adjunct to, rather than a substitute for, established HDP prevention and management. Risk-appropriate low-dose aspirin, optimization of chronic hypertension and diabetes, renal assessment, smoking cessation, appropriate gestational weight gain, blood pressure surveillance, fetal growth monitoring, and timely recognition of maternal organ dysfunction remain the foundations of care [1,2,36].

## 6. Clinical and Public Health Significance

### 6.1. Implications for Individualized Obstetric Care

The principal clinical implication is that metformin should remain indication-based. It may be selected for metabolic treatment in women with GDM, T2DM, or selected PCOS phenotypes, whereas any possible reduction in hypertensive risk should be presented as an uncertain secondary benefit rather than an established indication [7,8,9,10,22,23,24,25,26,27,28,29,30,31,32]. Counseling should clearly distinguish established effects on glycemic control, gestational weight gain, insulin requirements, and treatment burden from unproven prevention of gestational hypertension or preeclampsia [8,22,23,24,25,32,38].

Phenotype-based care is more informative than diagnosis alone. Women with marked insulin resistance, obesity-related inflammation, excessive gestational weight gain, higher fasting glucose, and a predominantly late-onset hypertensive risk phenotype may differ substantially from women with chronic hypertension, nephropathy, autoimmune disease, fetal growth restriction, or early angiogenic imbalance [3,5,6,14,16,33,34,39]. In the latter group, maternal vascular disease or impaired placental reserve may dominate the clinical risk and requires intensified placenta-oriented surveillance regardless of metformin exposure.

The decision to initiate or continue metformin should therefore integrate the original metabolic indication, baseline glycemic severity, gestational age, renal function, maternal cardiovascular and hypertensive history, aspirin eligibility, fetal growth trajectory, and the likelihood of requiring supplemental insulin [24,27,33,34,39]. A reduction in maternal weight gain or average birthweight should not be interpreted as uniformly beneficial without considering LGA, SGA, placental function, and fetal growth velocity [19,20,24,38,39,40,41].

Shared decision-making should address oral administration, gastrointestinal tolerability, placental transfer, uncertainty regarding long-term offspring outcomes, the possibility of supplemental insulin, and the need for continued blood pressure and fetal growth monitoring [14,16,17,18,19,20,21,33,38,40,41]. Metformin use should never reduce vigilance for symptoms, blood pressure changes, proteinuria, laboratory abnormalities, fetal growth restriction, or other clinical features of preeclampsia.

Women requiring supplemental insulin should not be regarded simply as metformin treatment failures. Higher body mass index, greater fasting hyperglycemia, and earlier treatment initiation predict the need for combined treatment and may identify a more severe metabolic phenotype [33,34]. Prompt escalation of therapy should therefore be part of individualized care rather than delayed in an attempt to preserve metformin monotherapy.

Metformin should also be positioned as an adjunct to established obstetric prevention and surveillance. It should not replace risk-appropriate low-dose aspirin, optimization of chronic hypertension and diabetes, renal assessment, regular blood pressure measurement, laboratory evaluation when indicated, or maternal–fetal surveillance [1,2,36].

### 6.2. Health System and Equity Considerations

HDP and diabetes in pregnancy represent major and unequally distributed global cardiometabolic burdens. Their frequency and consequences are influenced not only by biological risk but also by poverty, health literacy, delayed access to antenatal care, fragmented referral pathways, medication availability, diagnostic capacity, and broader social determinants of health [42,43]. Women in low- and middle-income settings experience a disproportionate burden of maternal and perinatal complications, while health systems often have fewer resources for early recognition, monitoring, and timely treatment.

A contemporary scoping review of HDP management in low- and middle-income countries identified recurrent barriers including limited availability of essential medications, unavailable diagnostic tests, deficiencies in healthcare infrastructure, shortages of trained personnel, restricted continuing professional education, outdated local protocols, and community misconceptions regarding preeclampsia [42]. These barriers reduce the likelihood that women will receive timely diagnosis, antihypertensive treatment, magnesium sulfate, appropriate referral, and medically indicated delivery.

Within this context, an inexpensive oral glucose-lowering medication is understandably attractive. Metformin can improve the feasibility of metabolic treatment where insulin storage, injection training, specialist review, frequent dose adjustment, or reliable access to consumables is difficult. Its affordability, oral administration, and lower treatment burden may therefore provide genuine public health value when it is used for an established GDM or T2DM indication [32,35,38,43].

However, medication accessibility alone cannot substitute for a comprehensive HDP strategy. Effective prevention and management require reliable antenatal blood pressure measurement, proteinuria and laboratory assessment, aspirin access for eligible women, emergency antihypertensive treatment, magnesium sulfate, trained personnel, referral capacity, and timely delivery [1,2,42]. A low-cost oral drug should not be promoted as a substitute for these health system functions.

Public health messaging must therefore avoid two opposite errors: underuse of metformin where it is an appropriate and accessible metabolic treatment, and overextension of metformin as a presumed low-cost solution for HDP prevention. The former may unnecessarily limit treatment options, while the latter may create false reassurance and divert attention from proven surveillance and emergency care pathways.

Implementation pathways should include clear eligibility criteria, assessment of renal function and contraindications, glucose monitoring, predefined treatment escalation thresholds, and guaranteed access to insulin when oral therapy is insufficient [33,34,35]. Systems should also document aspirin use, chronic hypertension, renal disease, fetal growth concerns, and referral thresholds so that metabolic treatment does not become isolated from broader obstetric risk management.

Equity should additionally be considered in research design. Trials conducted predominantly in highly resourced specialist centers may not capture adherence, access, monitoring, and referral challenges encountered in routine practice. Future studies should include geographically, ethnically, and socioeconomically diverse populations and assess implementation outcomes alongside efficacy and safety [42,43].

### 6.3. Postpartum Cardiovascular and Metabolic Prevention

The public health relevance of this topic extends well beyond the index pregnancy. HDP, GDM, T2DM, obesity, and PCOS identify women with overlapping risks of chronic hypertension, type 2 diabetes, ischemic heart disease, heart failure, stroke, and other cardiovascular outcomes [43,44,45]. Pregnancy can therefore be viewed as a cardiovascular and metabolic stress test that reveals pre-existing or latent susceptibility years before conventional risk assessment would otherwise identify it.

A systematic review and meta-analysis including more than 1.2 million women with previous HDP found substantially increased later risks of chronic hypertension, ischemic heart disease, and heart failure, with the risk of hypertension particularly high during the first five years after delivery [44]. These findings support early postpartum assessment rather than postponing cardiovascular prevention until symptoms develop.

HDP should consequently be regarded not only as acute obstetric complications but also as sex-specific cardiovascular risk markers. The postpartum period provides an opportunity to identify persistent hypertension, dysglycemia, obesity, dyslipidemia, smoking, renal dysfunction, and other modifiable risk factors while women are still connected to maternity services [44,45].

The maternal–fetal dyad extends this prevention framework across generations. Maternal hyperglycemia, obesity, hypertension, inflammation, placental dysfunction, and pharmacological exposure may influence fetal cardiovascular and metabolic development through developmental programming [18,19,20,21,38,40,41,43,46]. These effects may alter growth trajectories, adiposity, vascular function, blood pressure, glucose regulation, and later cardiometabolic susceptibility, although genetic, postnatal, environmental, and socioeconomic factors also contribute.

Current offspring evidence after metformin exposure is generally reassuring regarding neurodevelopment, later body mass index, metabolic markers, and childhood adiposity, but follow-up remains heterogeneous and incomplete [19,20,21,38,40,41]. Pregnancy interventions should therefore be evaluated not only for short-term maternal glycemia or neonatal size but also for longitudinal outcomes in both the mother and the child.

Postpartum care should include early blood pressure reassessment, evaluation for persistent hypertension, postpartum glucose testing after GDM, weight and lifestyle support, and lipid, renal, or broader cardiovascular assessment when clinically appropriate [44,45]. Women with severe, recurrent, or preterm preeclampsia; chronic hypertension; nephropathy; or multiple cardiometabolic risk factors may require more intensive and earlier follow-up.

Structured transition from obstetric to primary care, endocrinology, or cardiology services is essential. Personalized postpartum pathways, home blood pressure monitoring, dedicated postpartum cardiovascular clinics, patient education, and improved communication between obstetric and non-obstetric teams have been proposed to reduce the current loss to follow-up [45]. Nevertheless, implementation remains inconsistent, and guideline recommendations concerning long-term cardiovascular surveillance vary in quality and detail [47].

Metformin may have a separate role in postpartum diabetes prevention or long-term metabolic management in selected women, particularly after GDM or in established T2DM. This role should remain conceptually distinct from the hypothesis that antenatal metformin prevents HDP. Maintaining that distinction avoids extrapolating postpartum metabolic efficacy into an unproven obstetric preventive indication.

### 6.4. Implications for Policy and Research Prioritization

For policy makers, the appropriate priority is not routine metformin prophylaxis for HDP but the development of integrated pathways for metabolic and hypertensive pregnancy risk. Such pathways should combine preconception counseling, early risk assessment, glycemic management, aspirin prophylaxis when indicated, blood pressure surveillance, access to emergency treatment, fetal monitoring, referral, and structured postpartum prevention [1,2,42,43,44,45,46,47].

Clinical registries and trials should capture the indication for metformin, gestational age at initiation, dose, adherence, treatment discontinuation, supplemental insulin requirement, achieved glycemic control, aspirin exposure, chronic hypertension, renal disease, maternal vascular complications, fetal growth outcomes, and standardized HDP diagnoses [33,34,35,36,37]. Without these variables, it remains difficult to distinguish pharmacological effects from maternal phenotype, treatment selection, and health system context.

Future cost-effectiveness analyses should compare complete care pathways rather than drug acquisition costs alone. The relevant comparison is not simply oral metformin versus injectable insulin, but indication-appropriate therapy combined with monitoring, escalation, education, laboratory assessment, referral, and postpartum follow-up. A medication that is inexpensive but used without adequate monitoring may not be cost-effective when downstream maternal or neonatal complications are considered.

This broader health-economic perspective is particularly important in resource-constrained settings. Metformin may reduce injection-related costs and treatment burden, but any economic benefit depends on timely recognition of insufficient glycemic control, access to insulin when required, and preservation of essential HDP services [33,35,42,43].

Policy should also support standardized postpartum cardiovascular prevention. Despite strong epidemiological evidence linking HDP with future cardiovascular disease, guideline recommendations for long-term follow-up remain inconsistent, and many women do not receive structured counseling or risk factor surveillance [44,45,46,47]. Clear accountability for transition from maternity to primary care is therefore needed.

Research prioritization should move toward a precision prevention framework in which maternal metabolic phenotype, cardiovascular and renal status, placental biomarkers, gestational timing, aspirin exposure, and treatment response are evaluated as potential effect modifiers. Trials should distinguish gestational hypertension, preterm preeclampsia, term preeclampsia, severe disease, and superimposed preeclampsia rather than relying solely on composite HDP outcomes [2,3,14,16,26,35,37].

Long-term maternal and offspring follow-up should be incorporated into funding and trial infrastructure from the outset. Short-term metabolic benefit is insufficient to establish net public health value when both pregnancy complications and intrauterine exposures may influence health across the life course [38,40,41,44,45].

Until phenotype-specific benefit is demonstrated, metformin’s public health value lies primarily in accessible and effective metabolic treatment and in supporting broader cardiometabolic risk reduction pathways. It should not be positioned as a replacement for established HDP prevention, maternal surveillance, emergency treatment, or postpartum cardiovascular care. A phenotype-informed framework for the design and reporting of future studies is presented in Table 3.

## 7. Evidence Gaps and Future Research Priorities

The available literature is substantially stronger for the metabolic effects of metformin than for its effects on hypertensive outcomes. Most pregnancy trials were designed primarily to evaluate glycemic control, gestational weight gain, insulin requirements, neonatal size, or composite perinatal outcomes. Gestational hypertension and preeclampsia were frequently secondary or exploratory endpoints, with relatively few events and insufficient statistical power to detect modest but clinically relevant treatment effects [7,8,9,10,22,23,24,25,26,27,28,29,30,31]. Consequently, both favorable and neutral HDP estimates remain vulnerable to imprecision.

Future trials should prespecify clinically meaningful and standardized hypertensive outcomes. Gestational hypertension, preeclampsia without severe features, preeclampsia with severe features, superimposed preeclampsia, eclampsia, and HELLP syndrome should be reported separately, together with gestational age at diagnosis and delivery [2,3,37]. The international core outcome set for preeclampsia research identifies 14 maternal and eight offspring outcomes that should underpin future randomized trials and systematic reviews [48].

Relevant maternal outcomes include death, eclampsia, stroke, pulmonary edema, acute kidney injury, liver complications, placental abruption, postpartum hemorrhage, raised liver enzymes, thrombocytopenia, intensive care admission, and respiratory support [48]. Relevant offspring outcomes include stillbirth, gestational age at delivery, birthweight, SGA, neonatal death, seizures, neonatal unit admission, and respiratory support [48]. Incorporation of these outcomes would reduce selective reporting and improve comparability across trials.

Outcome harmonization is equally important in diabetes-specific research. The core outcome set for GDM prevention and treatment includes HDP, adherence, gestational weight gain, need and type of pharmacological therapy, mode of birth, birthweight, LGA, SGA, preterm birth, neonatal hypoglycemia, stillbirth, and neonatal death [49]. The corresponding core outcome set for pregestational diabetes specifically distinguishes pregnancy-induced hypertension from preeclampsia and includes trimester-specific HbA1c, maternal weight gain, severe hypoglycemia, diabetic ketoacidosis, fetal growth outcomes, congenital anomalies, stillbirth, and neonatal mortality [50]. Future metformin trials should align their outcome selection with both the preeclampsia and diabetes core outcome sets [48,49,50].

Phenotypic heterogeneity remains another major limitation. GDM, pregestational T2DM, obesity without diabetes, and PCOS differ in baseline cardiovascular risk, insulin resistance, β-cell reserve, renal disease, chronic hypertension, placental vulnerability, and timing of metformin exposure [5,6,22,23,24,25,26,27,28,29,30,31]. Even within a single diagnosis, women who respond to metformin monotherapy may differ substantially from those who require supplemental insulin [33,34].

Future studies should therefore recruit more homogeneous populations or prespecify stratified analyses according to maternal indication, pre-pregnancy body mass index, fasting glucose, HbA1c, gestational age at diagnosis, previous HDP, chronic hypertension, nephropathy, insulin requirement, and fetal growth status. Phenotype-enriched trials may be more informative than larger but biologically heterogeneous studies.

Timing and intensity of exposure are also incompletely characterized. Metformin initiated after conventional GDM diagnosis addresses a different biological question from treatment beginning before conception or during the first trimester. Later treatment may predominantly affect maternal glycemia and gestational weight gain, whereas earlier exposure could theoretically influence placental adaptation but also prolong direct fetal exposure [3,14,16,17,18,19,22,24,30].

Trials should report gestational age at initiation, starting and maximum dose, cumulative exposure, adherence, gastrointestinal intolerance, discontinuation, renal function, and the timing and dose of supplemental insulin. Direct comparisons of earlier and later initiation would be informative only if maternal indication, baseline metabolic severity, and background preventive care are standardized.

Mechanistic uncertainty remains substantial. Clinical associations cannot determine whether a possible HDP effect is mediated through improved maternal metabolism, reduced gestational weight gain, altered inflammatory signaling, enhanced endothelial function, or direct placental actions [12,13,15]. Biomarker improvement should not be equated with clinical prevention, particularly when experimental models, drug concentrations, and exposure durations differ from those encountered in pregnancy.

Mechanistically informed trials should combine validated metabolic indices with serial blood pressure assessment, endothelial and inflammatory biomarkers, angiogenic markers, and fetal growth measures. Potential mediators and effect modifiers could include fasting insulin, HOMA-IR where appropriate, lipid profile, C-reactive protein, inflammatory cytokines, endothelial markers, sFlt-1, PlGF, and the sFlt-1/PlGF ratio [12,13,14,15,16]. Placental histopathology and uterine or umbilical artery Doppler assessment may further distinguish metabolic–endothelial disease from predominantly placenta-mediated pathology.

Standard preventive treatment is an additional source of confounding and effect modification. Aspirin eligibility, dose, gestational age at initiation, adherence, and balance between study groups are inconsistently reported, despite their potential to alter the absolute risk of preeclampsia and the apparent incremental effect of metformin [1,2,36]. Chronic antihypertensive treatment, calcium supplementation where relevant, smoking, lifestyle intervention, and maternal weight management may produce similar analytical problems.

Future trials should standardize or carefully document these co-interventions. Aspirin use should be prespecified, reported by dose and timing, and included in stratified or interaction analyses where statistical power permits. A metformin effect observed against inconsistent background prevention would otherwise remain difficult to interpret.

Offspring safety is a further research priority because metformin crosses the placenta and directly exposes fetal and placental tissues [14,16,17,18]. Available evidence is generally reassuring regarding congenital anomalies, short-term neonatal outcomes, neurodevelopment, later childhood adiposity, and metabolic measures, but follow-up remains heterogeneous and frequently affected by attrition [19,20,21,38,40,41].

Future studies should prospectively follow offspring through childhood and adolescence rather than relying on opportunistic follow-up of the original trial population. Relevant outcomes include longitudinal growth, body composition, blood pressure, vascular function, glucose and insulin metabolism, lipid profile, pubertal development, neurodevelopment, and cardiovascular markers [19,20,21,38,40,41]. Maternal indication, comparator treatment, glycemic control, fetal growth phenotype, and postnatal environment should be incorporated into the analysis.

Individual participant data meta-analysis may help address several of these limitations. Harmonized participant-level data could standardize HDP definitions, classify timing and severity, distinguish metformin monotherapy from combined treatment, and evaluate interactions with maternal phenotype, aspirin use, renal disease, and gestational age at initiation [33,34,35,36,37,47,48,49]. However, such analyses will remain limited unless future trials collect comparable variables and adopt standardized core outcomes.

The priority is therefore not simply to increase the number of metformin studies, but to improve their biological specificity, outcome standardization, statistical power, and long-term follow-up. Only adequately powered, phenotype-informed trials can determine whether metformin provides a clinically meaningful secondary vascular benefit in selected women without compromising placental function, fetal growth, or offspring health. The principal evidence gaps and corresponding research priorities are summarized in Table 4.

## 8. Conclusions

Metformin is an important metabolic therapy in selected pregnancies, but its role in the prevention of hypertensive disorders of pregnancy remains incompletely defined. The biological rationale is compelling because insulin resistance, obesity-related inflammation, oxidative stress, endothelial dysfunction, mitochondrial disturbance, and placental angiogenic imbalance provide plausible links between maternal metabolic disease and the development of gestational hypertension and preeclampsia. However, mechanistic plausibility should not be equated with demonstrated clinical effectiveness.

Clinical evidence remains heterogeneous. Some meta-analyses have reported lower pregnancy-induced hypertension or preeclampsia, particularly when metformin was compared with insulin or when clinically heterogeneous populations were pooled. In contrast, contemporary randomized trials and large population-based analyses in women with GDM, T2DM, obesity, or PCOS have not demonstrated a consistent reduction in gestational hypertension, preeclampsia, composite HDP, or preterm preeclampsia.

This inconsistency is more compatible with a modest, phenotype-dependent, and predominantly indirect vascular effect than with a universal anti-preeclampsia action. Metformin may be more likely to modify hypertensive risk where insulin resistance, excessive gestational weight gain, obesity-related inflammation, and maternal metabolic–endothelial dysfunction are prominent. It is less likely to reverse severe placental maldevelopment or profound angiogenic imbalance once these processes are established. Nevertheless, this distinction between metabolically enriched and predominantly placenta-mediated disease remains a hypothesis requiring prospective validation.

Metformin should therefore continue to be prescribed for established metabolic indications rather than specifically for HDP prevention. It should not replace low-dose aspirin prophylaxis when indicated, optimization of chronic hypertension and diabetes, renal assessment, blood pressure surveillance, recognition of maternal organ dysfunction, or appropriate maternal–fetal monitoring. Its use should be individualized according to maternal indication, metabolic severity, renal and vascular status, gestational age, fetal growth trajectory, tolerability, and the likelihood of requiring supplemental insulin.

The benefit–risk assessment should also account for placental transfer and fetal exposure. Available evidence is broadly reassuring regarding congenital anomalies, short-term neonatal outcomes, childhood neurodevelopment, and later adiposity or metabolic outcomes, but follow-up remains heterogeneous and incomplete. Particular caution is warranted in pregnancies complicated by chronic hypertension, nephropathy, maternal vascular disease, fetal growth restriction, or suspected placental insufficiency, in which lower neonatal size may represent fetal undernutrition rather than a favorable reduction in pathological overgrowth.

Future trials should recruit clearly defined maternal populations and be adequately powered for prespecified, standardized hypertensive outcomes. Gestational hypertension, preeclampsia with and without severe features, superimposed preeclampsia, eclampsia, HELLP syndrome, and disease requiring delivery before 34 or 37 weeks should be reported separately. Studies should document gestational age at metformin initiation, dose, adherence, supplemental insulin, achieved glycemic control, aspirin exposure, chronic hypertension, renal disease, and fetal growth outcomes.

Mechanistically informed research should incorporate metabolic, endothelial, inflammatory, and angiogenic phenotyping, including sFlt-1, PlGF, and the sFlt-1/PlGF ratio, while recognizing that these biomarkers are not currently validated for selecting women for metformin treatment. Prospective long-term assessment of offspring growth, adiposity, blood pressure, vascular function, glucose and lipid metabolism, neurodevelopment, and pubertal outcomes should form an integral component of future benefit–risk evaluation.

Overall, metformin should be regarded as an indication-based metabolic treatment with a possible but unproven secondary vascular benefit in selected maternal phenotypes. Its clinical and public health value currently lies in improving accessible metabolic care and supporting integrated cardiometabolic risk reduction, rather than in replacing established strategies for the prevention, detection, and management of hypertensive disorders of pregnancy.

## Figures and Tables

**Table 1:** Biological pathways linking metformin with hypertensive disorders of pregnancy.

Pathway	Role in HDP	Potential Metformin Effect	Interpretation
Insulin resistance and hyperinsulinemia	Excessive insulin resistance contributes to dyslipidemia, oxidative stress, inflammation, sympathetic activation, sodium retention, and endothelial dysfunction. These abnormalities are common in GDM, T2DM, obesity, and PCOS and are associated with increased HDP risk [5,6,12].	Improves insulin sensitivity, reduces hepatic glucose production, and may decrease the maternal metabolic burden contributing to endothelial stress [7,8,12].	Most relevant to metabolically enriched phenotypes; improvement in insulin resistance does not necessarily imply correction of an established placental lesion.
Endothelial dysfunction and impaired nitric oxide signaling	Reduced nitric oxide bioavailability, increased vascular resistance, oxidative injury, and impaired vasodilatory responsiveness are central features of the maternal syndrome of preeclampsia [3,12].	AMPK activation may enhance eNOS activity and nitric oxide bioavailability, reduce endothelial inflammatory activation, and improve vasodilatory responsiveness [12,15]. Experimental human vessel studies showed improved vasodilation and reduced endothelial dysfunction after metformin exposure [12].	Supports a potential vascular benefit, but clinical confirmation through adequately powered HDP trials remains insufficient [7,8,9,10].
Oxidative stress and mitochondrial dysfunction	Placental hypoxia, excessive reactive oxygen species, and mitochondrial dysfunction contribute to trophoblast injury, angiogenic imbalance, and maternal endothelial dysfunction [3,12].	Metformin alters mitochondrial complex I activity and cellular energy metabolism. In trophoblast models, supratherapeutic concentrations impaired oxidative metabolism and differentiation, whereas therapeutic-range concentrations had limited effects [15]. In primary human trophoblasts, clinically relevant concentrations reduced mitochondrial respiration and oxidative stress markers [18].	Effects may be protective under excessive oxidative stress, but reduced placental ATP production raises questions regarding fetal growth and placental energy availability [18].
Inflammatory activation	Maternal obesity, diabetes, and preeclampsia are associated with increased inflammatory cytokines, adipokine imbalance, and NF-κB activation, which may amplify endothelial and placental dysfunction [5,6,13,15].	Metformin inhibits NF-κB-related signaling and has been associated with lower CRP, TNF-α, IL-1, and IL-6 concentrations in experimental and clinical biomarker studies [13,15].	Anti-inflammatory effects are biologically plausible but have not been shown consistently to translate into lower clinical HDP rates [7,8,9,10,13].
Placental and angiogenic dysfunction	Abnormal trophoblast invasion, impaired spiral artery remodeling, placental malperfusion, and excess release of sFlt-1 and soluble endoglin contribute to angiogenic imbalance and maternal endothelial injury [3].	Metformin reduced sFlt-1 and soluble endoglin secretion from primary endothelial cells, cytotrophoblasts, and preeclamptic placental explants and improved vasodilation and angiogenesis in experimental human tissues [12]. A systematic review confirmed reductions in antiangiogenic and inflammatory biomarkers in experimental and biomarker studies [13].	Supports a possible direct placental–vascular action, but experimental biomarker improvement should not be equated with proven prevention of preeclampsia [7,8,9,10,12,13].
sFlt-1/PlGF biomarker phenotype	The sFlt-1/PlGF ratio reflects angiogenic imbalance and is associated with placenta-mediated disease, disease severity, and short-term maternal and fetal risk [14]. In women with GDM, the ratio retains predictive value, although the metabolic disorder may influence biomarker concentrations [16].	Metformin reduces sFlt-1 and soluble endoglin secretion experimentally, but clinical effects on circulating sFlt-1, PlGF, or their ratio remain inconsistent or insufficiently studied [12,13,15].	A candidate stratification biomarker and possible effect modifier for future trials; it is not currently validated for selecting women for metformin treatment [14,16].
Gestational weight gain and adipose tissue dysfunction	Excessive gestational weight gain and obesity-related adipose dysfunction increase metabolic inflammation, insulin resistance, and hypertensive risk [5,7,8].	Metformin consistently reduces gestational weight gain in several trials and meta-analyses and may thereby reduce maternal metabolic and vascular load [7,8].	Lower gestational weight gain may partly explain favorable HDP estimates, but it should not be interpreted as direct prevention of placenta-mediated preeclampsia.
Placental transfer and fetal exposure	Metformin readily crosses the placenta, and fetal or cord-blood concentrations may approach maternal concentrations [17]. Matched maternal, fetal, and placental samples confirm substantial direct fetoplacental exposure [18].	Direct exposure may influence placental mitochondrial respiration, ATP production, AMPK–mTOR nutrient sensing, fetal growth, and postnatal body composition trajectories [18,19].	Exposure is acceptable for established metabolic indications, but it argues against expanding use solely for an unproven HDP prevention indication. Long-term offspring assessment remains necessary [18,19,20,21].
Fetal growth and developmental programming	Maternal hyperglycemia, obesity, placental dysfunction, and pharmacological exposure may influence fetal growth and later cardiometabolic development [17,18,19,20,21].	Metformin exposure is associated with slightly lower birthweight and selected anthropometric differences compared with insulin in some meta-analyses [19]. Long-term studies are generally reassuring but remain limited and heterogeneous [20,21].	Potential benefit from reducing pathological overgrowth must be balanced against possible SGA risk and uncertainty regarding long-term body composition, vascular, and metabolic outcomes [18,19,20,21].
Timing of treatment relative to placentation	Early placental development and spiral artery remodeling occur before metformin is initiated in most GDM trials; established placental maldevelopment may therefore be less modifiable by late treatment [3,7,10].	Earlier exposure in T2DM or PCOS could theoretically influence placental adaptation, whereas later exposure may mainly improve maternal metabolic physiology [8,9].	Timing should be treated as a potential effect modifier. Earlier use also increases the duration of fetal exposure [17,18,19,20,21].

**Table 2:** Clinical evidence on metformin and hypertensive outcomes according to maternal population.

Population	Main Evidence	HDP Findings	Clinical Interpretation	Gestational Age at HDP Onset Reported
Gestational diabetes mellitus (GDM)	Randomized trials, including EMERGE and SUGAR-DIP, population-based cohorts, and multiple conventional and network meta-analyses [7,8,9,10,22,23].	Some meta-analyses reported lower pregnancy-induced hypertension or preeclampsia with metformin than with insulin, although the direction of effect differed according to the specific HDP outcome [7,8,23]. EMERGE was not designed primarily to evaluate HDP, while SUGAR-DIP and the Scotland–Sweden cohort showed no material reduction in hypertensive outcomes [9,10,22].	Metformin may reduce metabolic contributors to hypertensive risk, including gestational weight gain and insulin resistance, in selected women. However, it is not an HDP-specific preventive therapy, and favorable pooled estimates may partly reflect comparator effects, treatment response, or differences in baseline metabolic severity.	Rarely reported. Most studies did not distinguish early- from late-onset preeclampsia or report gestational age at HDP onset systematically [7,8,9,10,22,23].
Pregestational T2DM or diabetes diagnosed early in pregnancy	Randomized trials of metformin added to insulin, including MiTy and MOMPOD; a planned MOMPOD analysis of preterm preeclampsia; population-based data; and target trial emulation [9,24,25,26,27].	MiTy found identical rates of hypertensive disorders in the metformin and placebo groups [24]. MOMPOD did not show an overall clinical benefit, and its secondary analysis found no reduction in preeclampsia requiring delivery before 37 or 34 weeks [25,26]. Population-based and target trial analyses were also largely neutral [9,27].	Metformin may improve glycemic control, reduce insulin requirements and gestational weight gain, and reduce excessive fetal growth, but these metabolic benefits have not translated consistently into lower HDP risk. Use should remain indication-based, with particular caution in women with nephropathy, chronic hypertension, vascular disease, or impaired placental reserve.	Usually not reported systematically. The MOMPOD secondary analysis specifically examined preeclampsia requiring delivery before 37 and before 34 weeks, but most other studies did not classify HDP by gestational age at onset [24,25,26,27].
Overweight or obesity without diabetes	The placebo-controlled GRoW and EMPOWaR trials and meta-analyses of randomized studies [7,8,28,29].	GRoW showed a modest reduction in weekly gestational weight gain but no improvement in major pregnancy outcomes [28]. EMPOWaR did not improve maternal or fetal outcomes [29]. Meta-analyses suggested a possible reduction in preeclampsia, but estimates were heterogeneous and based on relatively few trials [7,8].	Obesity alone is not an established indication for metformin during pregnancy. The evidence does not support routine use solely to limit gestational weight gain or prevent HDP. Lifestyle intervention, aspirin according to standard risk criteria, and blood pressure surveillance remain preferred.	Not systematically reported. GRoW, EMPOWaR, and the relevant meta-analyses did not provide consistent classification according to early- versus late-onset disease [7,8,28,29].
Polycystic ovary syndrome (PCOS)	PregMet2 and systematic reviews and meta-analyses, including analyses restricted to randomized controlled trials [30,31].	PregMet2 did not demonstrate a significant reduction in preeclampsia or pregnancy-induced hypertension, which were secondary outcomes [30]. Some broader pooled analyses suggested benefit, whereas meta-analysis restricted to randomized trials found no significant reduction in preeclampsia [31].	Metformin may be continued for selected metabolic or reproductive indications, but routine continuation throughout pregnancy solely to prevent HDP is not supported. Interpretation should account for obesity, insulin resistance, hyperandrogenism, infertility treatment, and preconception exposure.	Generally not reported. PregMet2 and randomized-trial meta-analyses did not distinguish HDP according to gestational age at onset or angiogenic phenotype [30,31].

**Table 3:** Proposed framework for future studies of metformin and hypertensive disorders of pregnancy.

Domain	Recommended Design or Measurement	Rationale
Maternal population	Study GDM, pregestational T2DM, obesity without diabetes, and PCOS separately; avoid pooling distinct indications in the primary analysis [7,8,9,10,24,25,26,27,28,29,30,31].	These populations differ in baseline HDP risk, reason and timing of metformin exposure, metabolic severity, vascular comorbidity, and duration of fetal exposure.
Baseline metabolic phenotype	Record pre-pregnancy BMI, gestational weight gain, fasting and post-load glucose, HbA1c, diabetes duration, insulin resistance markers, previous GDM, and gestational age at diagnosis [33,34].	These variables may predict metformin response, supplemental insulin requirement, and underlying metabolic severity.
Baseline vascular and renal phenotype	Record chronic hypertension, antihypertensive use, nephropathy, renal function, previous HDP, cardiovascular disease, and baseline blood pressure trajectory [24,27,39].	Vascular and renal disease may dominate HDP risk and may identify women at greater risk of SGA or placental insufficiency.
Timing of exposure	Separate preconception, first-trimester, early-pregnancy, and post-GDM-diagnosis initiation; report gestational age continuously and categorically [3,22,24,30].	Treatment initiated after placentation addresses a different biological mechanism from treatment initiated before or during early placental development.
Metformin exposure	Report starting dose, maximum dose, dose escalation, duration, adherence, gastrointestinal intolerance, treatment discontinuation, and renal-function-based modifications.	Variable exposure may partly explain heterogeneous efficacy and safety findings.
Comparator	Clearly distinguish insulin, placebo, usual care, lifestyle intervention, oral agent sequence, and continuation-versus-discontinuation designs [9,10,22,23,24,25,26,27,28,29,35].	Different comparators answer different causal questions and create different risks of confounding.
Treatment response	Report metformin monotherapy success, supplemental insulin requirement, time to insulin initiation, insulin dose, and achieved glycemic control [10,22,33,34].	Supplemental insulin identifies a clinically relevant post-randomization phenotype associated with baseline metabolic severity.
Aspirin and co-interventions	Report aspirin eligibility, screening method, dose, gestational age at initiation, adherence, and discontinuation; document antihypertensive, nutritional, and lifestyle interventions [1,2,36].	Background prophylaxis may alter absolute HDP risk and modify or obscure any incremental metformin effect.
Primary HDP outcome	Prefer a prespecified, clinically specific outcome such as preeclampsia requiring delivery before 37 weeks rather than an undifferentiated composite.	A specific outcome improves biological interpretation and avoids treating clinically unequal HDP subtypes as equivalent.
HDP classification	Report gestational hypertension, preeclampsia without severe features, severe preeclampsia, superimposed preeclampsia, eclampsia, and HELLP syndrome separately [2,3,35,37].	These conditions differ in pathophysiology, severity, maternal morbidity, and potential responsiveness to metabolic treatment.
Gestational age and severity	Report diagnosis and delivery before 34 weeks, before 37 weeks, and at term; record maternal organ dysfunction, antihypertensive treatment, magnesium sulfate, ICU admission, and medically indicated delivery [26,37].	Timing and severity are essential for distinguishing severe placenta-mediated disease from later metabolically enriched phenotypes.
Angiogenic and placental phenotype	Measure sFlt-1, PlGF, sFlt-1/PlGF ratio, uterine artery Doppler, and, where feasible, placental histopathology [12,13,14,15,16].	These measures may identify predominantly placenta-mediated disease and test whether the metformin effect varies by angiogenic phenotype.
Maternal metabolic outcomes	Report glycemia, HbA1c, gestational weight gain, insulin dose, hypoglycemia, lipid profile, and inflammatory or endothelial biomarkers [8,12,13,15,22,23,24,25].	These outcomes clarify whether any HDP effect is mediated through maternal metabolic improvement.
Fetal growth and placental reserve	Use serial biometry and growth velocity; report LGA, macrosomia, SGA, severe SGA, fetal growth restriction, and Doppler abnormalities [19,20,24,27,38,39].	Mean birthweight alone cannot distinguish beneficial reduction in overgrowth from adverse fetal undernutrition.
Neonatal outcomes	Report gestational age at birth, spontaneous versus indicated preterm delivery, stillbirth, neonatal hypoglycemia, NICU admission, respiratory morbidity, and neonatal anthropometry or body composition [19,22,23,24,25,38,40,41].	Neonatal outcomes provide a broader assessment of both metabolic benefit and placental or fetal safety.
Long-term offspring follow-up	Assess growth trajectories, adiposity, blood pressure, glucose and lipid metabolism, puberty, neurodevelopment, and cardiovascular markers into adolescence [19,20,21,38,40,41].	Existing evidence is reassuring but heterogeneous and insufficient to exclude smaller long-term cardiometabolic effects.
Analysis strategy	Use intention-to-treat analysis as primary; prespecify interaction analyses by phenotype, treatment timing, aspirin exposure, and supplemental insulin; consider individual participant data meta-analysis [33,34,35].	This preserves randomization while allowing exploration of clinically plausible effect modifiers.
Equity and implementation	Include ethnically and socioeconomically diverse populations and report treatment access acceptability, adherence, and cost.	Metformin’s oral administration and lower treatment burden may be particularly relevant in settings where insulin access or safe use is difficult.

**Table 4:** Principal evidence gaps and research priorities for metformin and hypertensive disorders of pregnancy.

Evidence Gap	Current Limitation	Research Priority
HDP as a secondary outcome	Most metformin trials were designed for glycemic, anthropometric, or neonatal outcomes and were underpowered for gestational hypertension, preeclampsia, and severe maternal complications [7,8,9,10,22,23,24,25,26,27,28,29,30,31].	Conduct adequately powered trials with prespecified HDP endpoints and adopt the international preeclampsia core outcome set [47]. Report gestational hypertension, preeclampsia, severe features, superimposed preeclampsia, eclampsia, and HELLP syndrome separately.
Inconsistent outcome selection and reporting	Diabetes and obstetric trials use different maternal, fetal, and neonatal outcomes, limiting comparison and evidence synthesis [35].	Align outcome reporting with the core outcome sets for preeclampsia, GDM, and pregestational diabetes [47,48,49]. Report both maternal and offspring outcomes and avoid relying exclusively on composite endpoints.
Phenotypic heterogeneity	Metabolic, vascular, renal, and placental risk differs across GDM, T2DM, obesity, and PCOS and within each diagnostic group [5,6,22,23,24,25,26,27,28,29,30,31].	Conduct diagnosis-specific and phenotype-enriched trials. Prespecify stratification by BMI, glycemic severity, chronic hypertension, nephropathy, previous HDP, insulin resistance, and fetal growth phenotype.
Metformin response and supplemental insulin	Women requiring supplemental insulin may have greater fasting hyperglycemia, β-cell dysfunction, obesity, or earlier-onset metabolic disease than metformin-only responders [10,22,33,34].	Report metformin monotherapy success, timing of insulin escalation, insulin dose, and achieved glycemic control. Retain intention-to-treat analysis while adding carefully adjusted responder analyses.
Treatment timing and dose	Exposure windows, dose escalation, cumulative exposure, adherence, and duration of fetal exposure vary widely [14,16,17,18,19,22,24,30].	Compare clearly defined initiation windows and report gestational age at initiation, starting and maximum dose, cumulative exposure, adherence, discontinuation, renal function, and supplemental insulin.
Mechanistic uncertainty	Clinical outcomes do not establish whether observed effects are mediated through maternal metabolism, gestational weight gain, endothelial function, inflammation, or direct placental pathways [12,13,15].	Integrate validated insulin resistance indices, metabolic and endothelial markers, inflammatory pathways, and serial clinical outcomes. Use mediation analyses only when temporally appropriate measurements are available.
Angiogenic and placental phenotype	Few trials assess whether treatment effects vary according to placental dysfunction or angiogenic imbalance. The sFlt-1/PlGF ratio is not validated for selecting women for metformin [14,16].	Prospectively measure sFlt-1, PlGF, the sFlt-1/PlGF ratio, uterine artery Doppler indices, fetal growth velocity, and placental histopathology. Evaluate these measures as effect modifiers rather than treatment selection tests.
Confounding by comparator	Comparisons with insulin, placebo, lifestyle intervention, usual care, or treatment discontinuation answer different causal questions [9,10,22,23,24,25,26,27,28,29,35].	Match the comparator to the clinical question and avoid combining fundamentally different comparator groups in pooled analyses without stratification.
Confounding by standard prevention	Aspirin eligibility, dose, timing, adherence, and balance between groups are inconsistently reported [1,2,36]. Other co-interventions are also variable.	Prespecify and stratify by aspirin exposure, standardize background care where possible, adjust for chronic hypertension and renal disease, and test metformin-by-aspirin interaction when adequately powered.
Inadequate classification of HDP timing and severity	Early- and late-onset disease, preterm and term preeclampsia, and severe and non-severe phenotypes are frequently pooled [2,3,26,37].	Report diagnosis and delivery before 34 weeks, before 37 weeks, and at term. Include severe features, maternal organ dysfunction, ICU admission, and medically indicated preterm delivery.
Fetal growth interpretation	Lower mean birthweight may represent reduced pathological overgrowth or adverse fetal undernutrition; many studies do not report the full growth distribution [19,20,24,27,39].	Report serial growth, growth velocity, LGA, macrosomia, SGA, severe SGA, fetal growth restriction, Doppler findings, and placental pathology rather than mean birthweight alone.
Offspring safety	Direct fetal exposure occurs, but long-term metabolic, cardiovascular, growth, pubertal, and neurodevelopmental evidence remains limited and heterogeneous [14,16,17,18,19,20,21,38,40,41].	Establish prospective follow-up through childhood and adolescence, including growth, adiposity, blood pressure, vascular function, metabolism, neurodevelopment, and pubertal outcomes.
Maternal long-term outcomes	Trials focus on pregnancy and rarely evaluate whether maternal metabolic or cardiovascular effects persist after delivery.	Include postpartum blood pressure, persistent hypertension, glucose status, weight, lipid profile, renal function, cardiovascular risk factors, and subsequent pregnancies [44,45,46].
Limited external validity and equity	Many trials are conducted in specialist settings and may not reflect medication access, insulin availability, monitoring capacity, or referral barriers in routine practice [42,43].	Recruit geographically, ethnically, and socioeconomically diverse populations and include implementation, acceptability, cost, and health system outcomes.
Limited ability to identify treatment-effect heterogeneity	Aggregate data meta-analyses cannot reliably evaluate interactions involving phenotype, timing, aspirin, renal disease, or supplemental insulin.	Develop harmonized individual participant data meta-analyses using standardized definitions and core outcomes [33,34,35,36,37,47,48,49].

## Data Availability

No new data were created or analyzed in this study. Data sharing is not applicable to this article.
